# Different Effects of Angiotensin II and Angiotensin-(1-7) on Vascular Smooth Muscle Cell Proliferation and Migration

**DOI:** 10.1371/journal.pone.0012323

**Published:** 2010-08-23

**Authors:** Feng Zhang, Yanhua Hu, Qingbo Xu, Shu Ye

**Affiliations:** 1 William Harvey Research Institute, Barts and The London School of Medicine and Dentistry, Queen Mary University of London, London, United Kingdom; 2 Department of Physiology and Pathophysiology, Health Science Center, Peking University, Beijing, China; 3 Cardiovascular Division, King's BHF Centre, King's College London, London, United Kingdom; Innsbruck Medical University, Austria

## Abstract

**Background:**

Angiotensin (Ang) II and Ang-(1-7) are two of the bioactive peptides of the rennin-angiotensin system. Ang II is involved in the development of cardiovascular disease, such as hypertension and atherosclerosis, while Ang-(1-7) shows cardiovascular protection in contrast to Ang II.

**Methodology/Principal Findings:**

In this study, we investigated effects of Ang II and Ang-(1-7) on vascular smooth muscle cell (SMC) proliferation and migration, which are critical in the formation of atherosclerotic lesions. Treatment with Ang II resulted in an increase of SMC proliferation, whereas Ang-(1-7) alone had no effects. However, preincubation with Ang-(1-7) inhibited Ang II-induced SMC proliferation. Ang II promoted SMC migration, and this effect was abolished by pretreatment with Ang-(1-7). The stimulatory effects of Ang II on SMC proliferation and migration were blocked by the Ang II receptor antagonist lorsartan, while the inhibitory effects of Ang-(1-7) were abolished by the Ang-(1-7) receptor antagonist A-799. Ang II treatment caused activation of ERK1/2 mediated signaling, and this was inhibited by preincubation of SMCs with Ang-(1-7).

**Conclusion:**

These results suggest that Ang-(1-7) inhibits Ang II-induced SMC proliferation and migration, at least in part, through negative modulation of Ang II induced ERK1/2 activity.

## Introduction

Vascular smooth muscle cell migration and proliferation are important processes in the development of restenosis after angioplasty and in the formation of atherosclerotic plaques [1], [2]. After angioplasty, SMCs migrate from media into intima, where they contribute to neointima formation and restenosis. This process is also an important feature in the development of de nova atherosclerotic lesions, in which fibrous cap is characterized by the accumulation of SMCs and SMC-derived extracellular matrix.

Angiotensin II, one of the major active components in the rennin-angiotensin system, plays an essential role in the pathogenesis of atherosclerosis [3]. Most of the pathophysiologic actions of Ang II are mediated by signal transduction through the AT1 receptor. The AT1 receptor, which contains 7 transmembrane helixes, is a member of superfamily of G protein-coupled receptors. This receptor mediates the effects of Ang II on vasoconstriction, proliferation, inflammation, coagulation and extracellular matrix remodeling.[4] During the development of atherosclerosis, Ang II, acting through AT1 receptor, induces vascular SMC growth and migration [1], [4].

An major Ang II-induced signaling pathway is activation of mitogen-activated protein kinases, including extracellular signal-regulated kinase (ERK1/2) [5], p38 mitogen-activated protein kinase (p38 MAPK) [6], and c-Jun N-terminal kinase (c-JNK) [7]. ERK1/2 has been reported to be a critical regulatory factor for Ang II-mediated growth and migration of vascular SMCs [5], [8]–[11], Inhibition of ERK1/2 decreases Ang II-induced vascular SMC proliferation and migration [5], [11].

Angiotensin-(1-7), another bioactive peptide of the rennin-angiotensin system, appears to exert cardiovascular protection in contrast to Ang II. Ang-(1-7), which can be converted from Ang II by ACE2, has vasodilator and anti-proliferative properties [12]. In addition, it has been shown that Ang-(1-7) attenuates ventricular hypertrophy and fibrosis in response to the hypertensive challenge by Ang II [13]. The effects of Ang-(1-7) are mediated via the MAS receptor, another G-protein-coupled, seven transmembrane protein [14]. The peptide has been shown to oppose many actions of Ang II [15], and to counterregulate Ang II-induced ERK1/2 activity [16]–[18].

There are studies showing the effect of Ang-(1-7) on vascular SMC proliferation [16], [19]. Nevertheless, the effect of Ang-(1-7) on vascular SMC migration has not been well investigated. There is evidence showing that intravenouse infusions of Ang-(1-7) attenuates neointimal formation after vascular injury and stent implantation in rats [20], [21], which could be in part due to effects of Ang-(1-7) on SMC migration into the neointima. In this study, we investigated possible effects of Ang-(1-7) on vascular SMC proliferation and migration. We found that Ang-(1-7) inhibited Ang II-induced vascular SMC proliferation and migration, and this inhibitory effect was via the MAS receptor. Furthermore, we found that Ang-(1-7) treatment suppressed Ang II-induced activation of ERK1/2 mediated signaling, a possible mechanism for its inhibitory effect on SMC proliferation and migration.

## Results

### Ang-(1-7) inhibits Ang II-induced SMC proliferation

**Figure 1:**
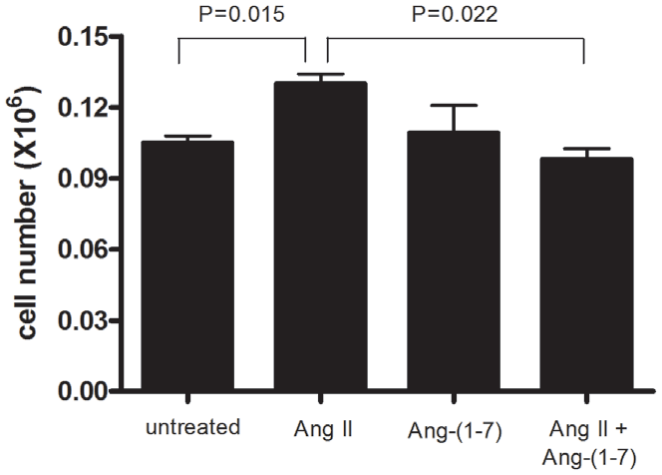
Ang-(1-7) inhibits Ang II-induced SMC proliferation. Data shown are mean ± SEM of cell numbers at day 4 from 4 experiments, each with triplicate wells per condition.

RT-PCR and subsequent sequence analysis showed that the SMCs expressed both the Ang II AT1 receptor and the Ang-(1-7) MAS receptor. To study effects of Ang II and Ang-(1-7) on SMC proliferation, same numbers of cells were seeded in individual wells of 6-well plates, cultured in the presence or absence of Ang II or Ang-(1-7) or both (Ang-(1-7) was added 10 min before the addition of Ang II) at a final concentration of 10^−7^ M for four days, and then the total numbers of cells per well were determined using a cell counter. As shown in Figure 1, there was a significantly greater increase in cell number when treated with Ang II, as compared with the untreated control. In contrast, treatment with Ang-(1-7) alone did not alter the rate of cell proliferation. However, when the cells were preincubated with Ang-(1-7), Ang II-induced SMC proliferation was significantly inhibited (Figure 1).

### MAS receptor antagonist abrogates the effects of Ang-(1-7) on SMC proliferation

**Figure 2:**
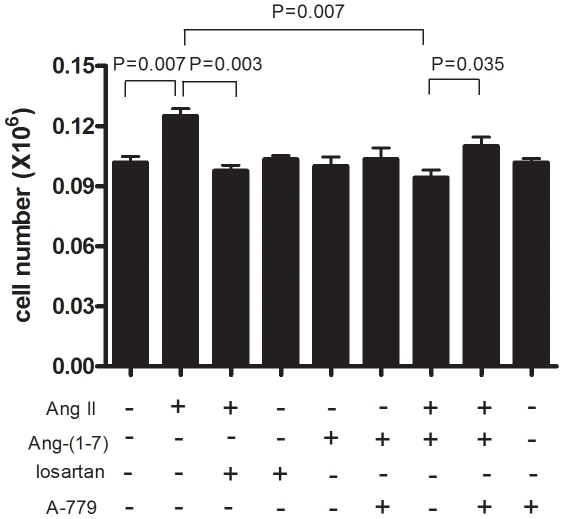
Antagonists abrogate the effects of Ang II and Ang-(1-7) on SMC proliferation. Data shown are mean ± SEM of cell numbers at day 4 from 4 experiments, each with triplicate wells per condition.

As Ang II mediates most of its effects via the AT1 receptor and Ang-(1-7) mediates its effects via the MAS receptor, we then used the AT1 receptor antagonist losartan and the MAS receptor antagonist A-779 to investigate whether Ang II and Ang-(1-7) exerted their effects on SMC proliferation via these two receptors respectively. As shown in Figure 2, the AT1 receptor antagonist losartan blocked Ang II-induced SMC proliferation, whilst the MAS receptor antagonist A-779 abrogated the anti-proliferative effect of Ang-(1-7) on Ang II-induced SMC proliferation.

### Ang-(1-7) modulates Ang II-stimulated SMC migration

**Figure 3:**
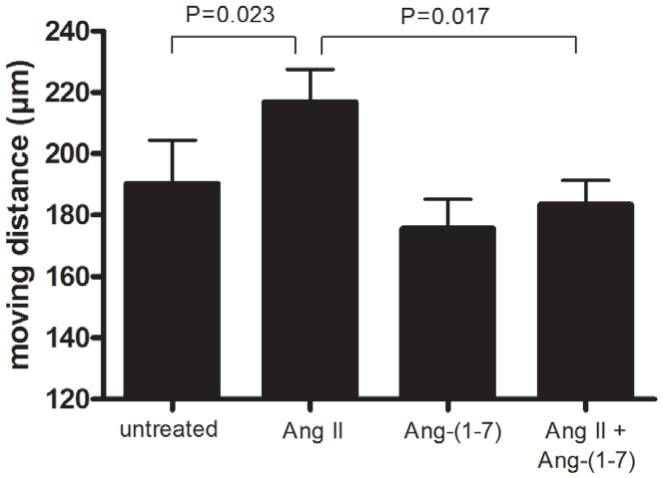
Ang-(1-7) inhibits the stimulating effect of Ang II on SMC migration. Data shown are mean ± SEM of migration distance from 4 experiments, each with triplicate wells per condition.

To study effects of Ang II and Ang-(1-7) on SMC migration, SMCs were subjected to migration assays, in the presences or absence of Ang II or Ang-(1-7) or both (Ang-(1-7) was added 10 min before the addition of Ang II). The experiments showed that Ang II increased SMC migration, whereas Ang-(1-7) alone had no effect (Figure 3). However, cells treated with both Ang II and Ang-(1-7) had significant lower rate of migration compared with cells treated with Ang II alone (Figure 3), suggesting that Ang-(1-7) inhibited the stimulating effect of Ang II on SMC migration.

### MAS receptor antagonist blocks the effect of Ang-(1-7) on SMC migration

**Figure 4:**
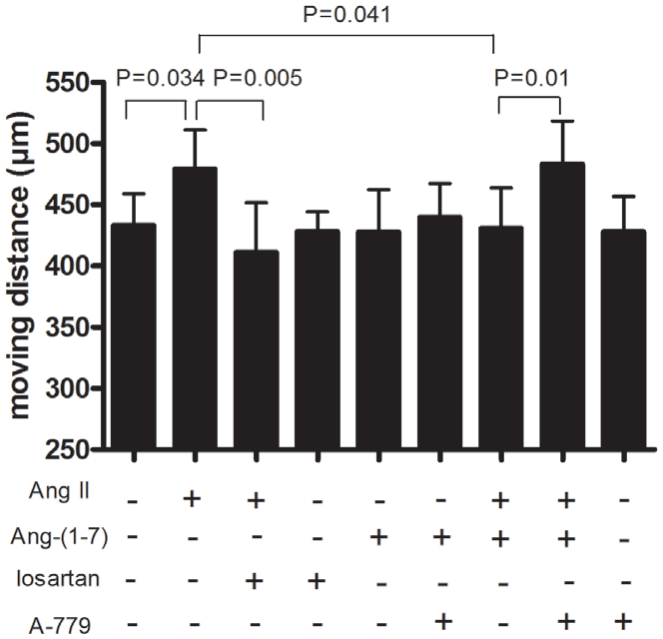
Blockade of the effects of Ang II and Ang-(1-7) on SMC migration by antagonists. Data shown are mean ± SEM of migration distance from 4 experiments, each with triplicate wells per condition.

To investigate whether the stimulating effect of SMC migration was mediated via the AT1 receptor and whether the counteracting effect of Ang-(1-7) was mediated by the MAS receptor, we performed the migration assays in the presence or absence of the AT1 receptor antagonist losartan or the MAS receptor antagonist A-779. As shown in Figure 4, Ang II-induced SMC migration was inhibited by AT_1_R antagonist losartan, while the inhibitory effect of Ang-(1-7) on Ang II-induced SMC migration was blocked by A-779.

### Ang-(1-7) inhibits AngII-induced ERK1/2 phosphorylation

**Figure 5:**
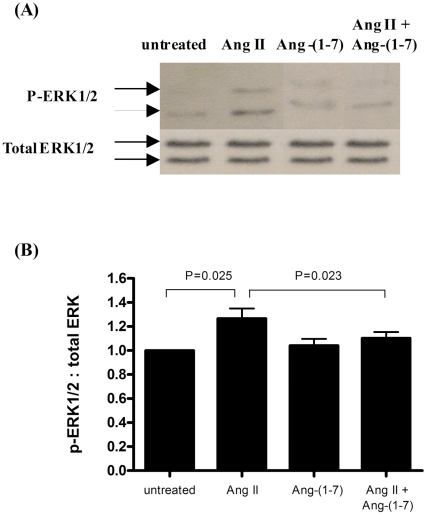
Ang-(1-7) inhibits Ang II-induced ERK1/2 phosphorylation. (A) Representative Western blotting results, showing bands for phosphorylated ERK1/2 (p-ERK1/2) and total ERK1/2. (B) Quantification of band intensity of Western blots. Data shown are mean ± SEM of 3 experiments.

Since the ERK1/2 signaling pathway plays a major role in Ang II-induced SMC proliferation and migration[5], [11], we investigated whether Ang-(1-7) interfered with Ang II-induced ERK1/2 activation. We found that Ang II treatment stimulated phosphorylation of ERK1/2 and that this effect was significantly inhibited in the presence of Ang-(1-7) (Figure 5).

## Discussion

Our study show that Ang-(1-7) and Ang II exert different effects on vascular SMC proliferation and migration. Our study confirms the reported stimulating effects of Ang II on SMC proliferation and migration, supporting the notion that increased Ang II generation can promotes atherogenesis and neointimal formation leading to restenosis after angioplasty [22]. Similar to previous studies which showed that Ang-(1-7) inhibits proliferation in a number of cell types [19], [23]-[25], we observed that Ang-(1-7) negatively modulates Ang II-induced vascular SMC proliferation. Besides, the most important finding of our study is that Ang-(1-7) inhibits Ang II-induced SMC migration. This novel finding provides a further rationale for the use of Ang-(1-7) as a therapeutic agent for preventing neointimal formation and restenosis after angioplasty [26], [27]. We found that the inhibitory effect of Ang-(1-7) on Ang II-stimulated SMC proliferation and migration can be blocked by the MAS receptor antagonist A-779, indicating that Ang-(1-7) exerts this effect via activating the MAS receptor. Furthermore, we show that Ang-(1-7) inhibits Ang II-induced ERK1/2 phosphorylation, which provides a plausible explanation for the molecular mechanism underlying the inhibitory effect of Ang-(1-7) on Ang II stimulated SMC proliferation and migration, since the ERK1/2 dependent signaling pathway plays a critical role in mediating the stimulating effect of Ang II on SMC proliferation and migration.

In our study, we observed that Ang-(1-7) inhibits Ang II-induced vascular SMC proliferation. Interestingly, whilst Ang II has been shown to induce proliferation of many cell types including vascular SMCs,[22] divergent effects have been reported for Ang-(1-7) in different cell types. It has been shown that Ang-(1-7) causes inhibition of proliferation of rat vascular SMCs, myocytes and human lung cancer cells,[19], [23], [24] and that Ang-(1-7) treatment attenuates neointimal formation after stent implantation in rats.[20] On the other hand, Ang-(1-7) simulates proliferation of endothelia progenitor cells and hematopoietic progenitor cells [28], [29]. Moreover, similar to our finding in mouse SMCs, Ang-(1-7) alone has no effect on mesangial cell growth, whereas Ang-(1-7) inhibits Ang II-induced increase in mesangial cell proliferation [25]. The reasons for these divergent effects of Ang-(1-7) are currently unknown and might be due to differences in the repertoires of receptors, signaling molecules and cell cycling regulators, etc, in the different types of cells. There is a reported study showing direct inhibition of rat vascular SMC proliferation by Ang-(1-7) [19], which differ from our finding in mouse SMCs, possibly because of the different animal specials and experiment conditions.

It has been well established that Ang II is a potent stimulus for vascular SMC migration [5], [6], [8], [10], [30]. In contrast, it was unclear whether Ang-(1-7) also had a effect on SMC migration, although there are several studies from which there is indirect evidence to suggest that this might be the case. Some of these studies showed that Ang-(1-7) treatment attenuated neointimal formation after vascular injury and stent implantation in the rat [20], [27]. Similarly, another study showed that Ang-(1-7) treatment resulted in a reduction of neointimal formation and collagen synthesis after angioplasty in rabbits [26]. As SMC migration is a critical step in the formation of neointima, it is possible that the reduction in neointimal formation as a result of Ang-(1-7) in these studies might be in part due to an inhibitory effect of Ang-(1-7) on SMC migration. The finding of our study that Ang-(1-7) inhibits Ang II-induced SMC migration provides direct evidence that supports this notion.

Our study shows that Ang II-induced ERK1/2 activation is negatively modulated by Ang-(1-7). Previous studies have shown that activation of ERK1/2 plays an important role in SMC migration. ERK1 and ERK2 are rapidly activated in rat carotid arteries after balloon injury [31]. Downregulation of ERK1/2 by antisense oligonucleotides or gene transfer of a dominant-negative mutant of ERK1/2 prevents neointimal formation in balloon angioplasty [32]. In addition, it has been shown that the effect of Ang II on vascular SMC migration are mediated via an AT1 receptor dependent signaling pathway involving activation of ERK1/2 [9], and that blocking ERK1/2 activation using the MAPK kinase inhibitor PD98059 or antisense oligodeoxynucleotides can significantly attenuate Ang II-stimulated SMC migration [5]. In the present study, we found that Ang-(1-7) reduced Ang II-stimulated ERK1/2 activity. It is likely that Ang-(1-7) can activate a signaling pathway that leads to blockage of ERK1/2 activation and therefore can inhibit Ang II- induced ERK activation and Ang II-stimulated SMC migration.

There are several possibilities for the mechanism by which Ang-(1-7) regulates SMC migration. Firstly, inhibition of the ERK1/2-dependant signally cascade by Ang-(1-7) is likely to play a role in reduced cell migration. Substrates of ERK1/2 include FAK (focal adhesion kinase) and paxillin [33], [34]. ERK1/2-dependant FAK and paxillin phosphorylation is important in the regulation of focal contacts during cell migration [35]. Ang II-induced activation of c-Src, another vital component in the regulation of focal contacts formation, has been shown to be abrogated by Ang-(1-7) in human endothelial cells, and c-Src is upstream of the ERK signaling cascade [18]. Ang-(1-7) antagonizes Ang II-mediated upregulation of matrix metalloproteinase (MMP)-9 [36], an extracellular matrix protein-degrading enzyme known to play an important role in cell migration [37]. MMP-9 expression has been shown to be mediated by ERK1/2, and inhibition of ERK1/2 downregulates MMP-9 expression in vascular SMCs and in arterialized vein grafts [38], [39]. As our study showed that Ang-(1-7) abrogated Ang II-mediated ERK1/2 activation, it is likely that Ang-(1-7) can counteract Ang II-induced SMC migration through inhibiting FAK and paxillin phosphorylation and MMP-9 expression via the ERK1/2 pathway. Secondly, Ang-(1-7) is a bioactive peptide that stimulates nitric oxide release [40], [41], and nitric oxide is known to inhibit Ang II-induced SMC migration [40], [42]. Thirdly, Ang-(1-7) can downregulate AT1 receptor, resulting in reduced Ang II binding in vascular SMCs [43].

The first evidence for an interaction between Ang-(1-7) and Ang II is the inhibition of the contractile effect of Ang II in the rabbit aorta by the Ang-(1-7) analogue Sar-Ang-(1-7) [44]. Further studies show that Ang-(1-7) antagonizes Ang II-induced contraction of human vessels, including internal mammary arteries and forearm resistant vessels [28], [45], [46]. Ang-(1-7) blockade of Ang II-induced contraction results from release of vasorelaxing factors including NO and prostaglandins [40], or other biologically active peptides such as bradykinin [47]. In addition, it has been shown that Ang-(1-7) prevents Ang II-induced cardiac remodeling, attenuating myocyte hypertrophy and myocardial interstitial fibrosis induced by Ang II [48]. The finding of our study that Ang-(1-7) inhibits the effect of Ang II on SMC proliferation and migration suggests that the interaction between Ang-(1-7) and Ang II could also occur in vascular remodeling via affecting SMC proliferation and migration.

The findings from the previous and present studies of different effects of Ang II and Ang-(1-7), which represent two major members of the rennin-angiotensin system, are directly relevant to understanding the pathogenesis of de nova atherosclerosis and neointimal formation after angioplasty. Moreover, the findings that Ang-(1-7) counteracts the atherogenic effect of Ang II have led to the development of therapeutic approaches utilizing the anti-atherogenic properties of Ang-(1-7), e.g. Ang-(1-7) coated stents [20]. The novel finding of the present study that Ang-(1-7) inhibits Ang II-induced SMC migration further supports the rationales for such therapeutic approaches.

## Materials and Methods

### Materials

Ang II, Ang-(1-7), losartan, Dulbecco's modified Eagle's medium (DMEM), penicillin and streptomycin were obtained from Sigma Aldrich. A-779 was purchased from Phoenix Pharmaceuticals. Fetal bovine serum (FBS) was obtained from Invitrogen. Antibodies to phospho-ERK1/2 and to total ERK1/2 were from Cell signaling.

### Cell culture

SMCs were isolated by autogrowth of explant culture from the thoracic aortas of mice as described before [49], which was approved by King's College Ethical Review Process Committee and under the UK Home Office Animal Scientific Procedures Division permit number 70/6458. Briefly, mouse thoracic aortas were removed and washed with DMEM. Intima and inner two thirds of media were carefully dissected from the vessels, cut into pieces (≈1 mm^3^). Tissue pieces were then explanted onto a 0.02% gelatin-coated flask. To get a firm attachment of tissue pieces, the flask was incubated upside-down for 1 h and then DMEM supplemented with 20% FBS, penicillin and streptomycin was slowly added. Cells were allowed to autogrow for 2 weeks and then passaged until enough cells were obtained. Cells were then maintained in DMEM supplemented with 10% FBS, penicillin and streptomycin at 37°C in a humidified atmosphere of 5% CO_2_.

### Reverse-transcriptase polymerase chain reaction (RT-PCR)

RNA was isolated from cultured SMCs using Nucleospin RNA II 50 Preps (Fisher Scientific) according to the manufacturer's instruction. Single-stranded cDNA was synthesized from 1 µg of total RNA using 0.5 µg random primers and 200 units of M-MLV reverse transcriptase (Promega). PCR of the AT1 receptor and the MAS receptor were carried out. The sequences of PCR primers for AT1-R were 5′-GCATCATCTTTGTGGTGGG-3′ (sense) and 5′-ATCAGCACATCCAGGAATG-3′ (anti-sense) as described elsewhere[50], which generated a 690 bp PCR product. The PCR primer sequences for the MAS receptor were, 5′-GGAACAGGACGGAGGTTACA-3′ (sense) and 5′-AGTCAGGAGGTGGAGAGCAA-3′ (anti-sense), which produced a 395 bp amplicon. The PCR products were analyzed by agarose gel electrophoresis to verify that they had the expected sizes and were subsequently further verified by sequencing.

### Measurement of cell proliferation

Some numbers of SMCs were seeded in different wells on 6-well tissue culture plates, cultured in the presence of 1% FBS and stimulated with Ang-(1-7) (10^−7^ M) or Ang II (10^−7^ M) or both (Ang-(1-7) was added 10 minutes before the addition of Ang II). In some conditions, cells were preincubated with losartan (10^−6^ M) or A-799 (10^−6^ M) for 5 minutes followed by treatment with Ang II or Ang-(1-7). The medium was changed and the cells were restimulted every 24 hours. After four days, cells were removed from each well using trypsin/EDTA and cell numbers were counted by a Vi-CELL cell viability analyzer (Beckham Counter).

### Migration assay

SMC migration was measured using a previously described method [51], [52]. Briefly, cells were grown to confluence on 6-well plates. Parallel lines were drawn with a maker pen on the back of each well. Liner wounds were made vertical to these parallel lines by scraping each well with a sterile 200-uL pipette tip. Cells were then rinsed twice with serum-free medium to remove cellular debris. Images besides each crossing of parallel lines and wounds were obtained using an Olympus inverted microscope equipped with a camera. Cells were incubated with or without losartan (10^−6^ M) or A-779 (10^−6^ M) for 5 minutes, then with or without the addition of Ang II (10^−7^ M) or Ang-(1-7) (10^−7^ M), or with the addition of Ang-(1-7) 10 minutes before addition of Ang II (10^−7^ M). After 24 hours, images were taken again besides each crossing. ImageJ software was used to measure the distance between two edges of each wound. Data are presented as moving distance, which is the difference in the distances between two edges at the same crossing at 0 h and 24 h.

### Western blotting

SMCs in serum-free media were incubated with or without Ang-(1-7) (10^−7^ M)for 5 minutes. Ang II (10^−7^ M) was then added and incubation was continued for an additional 30 minutes. Cell lysates were prepared in lysis buffer (1% SDS, 62.5 mM Tris-HCl, pH 7.8) containing protease inhibitor cocktail and phosphatase inhibitor cocktail (Sigma Aldrich) and protein concentrations were measured by a Bradford assay. Solubilized proteins (20 ug/well) were separated in 10% SDS-polyacrylamide gel by electrophoresis and transferred to nitrocellulose membranes. In the presence of 5% nonfat milk in Tris-buffered saline Tween 20 to block nonspecific binding, the membranes were hybridized overnight with an antibody for phosphorylated ERK1/2 (anti-phospho-ERK1/2, 1∶2000) or an antibody for total ERK1/2 (anti-total ERK1/2, 1∶1000), followed by incubation with a second antibody coupled to horseradish peroxidase. Immunoreactive bands were detected using a chemiluminescence (ECL) system and X-ray films. Densitometric analysis of bands was performed using Adobe Photoshop software.

### Statistical analyses

All results are expressed as mean ± SEM. The data were evaluated by paired t-test and confirmed by Wilcoxon test. A value of P<0.05 was considered significant.
